# The Homœopathic Law of Similarity

**Published:** 1888-01

**Authors:** 


THE HOMŒOPATHIC LAW OF SIMILARITY. By Dr. von Grauvogl. Translated by Dr. Geo. E. Shipman. Chicago, 1879.

If there be any one of our readers who has not read this "open letter of Dr. von Grauvogl to Baron von Liebig" then we advise him to do so at his earliest opportunity. Although Grauvogl did not accept Hahnemann's teachings as we do, nevertheless one cannot fail to gain many ideas from his forceful writing. This essay can be probably procured of any pharmacy.

